# UPLC–TOF–MS Method for Simultaneous Quantification of Steroid Hormones in Tissue Homogenates of Zebrafish with Solid-Phase Extraction

**DOI:** 10.3390/molecules26206213

**Published:** 2021-10-14

**Authors:** Yaxi Li, Zhi Yan, Xiaodong Li, Xiuli Yin, Ke Li

**Affiliations:** 1 Yantai Institute of Coastal Zone Research, Chinese Academy of Sciences, Yantai 264003, China; yxli@yic.ac.cn (Y.L.); yanzhistudy@s.ytu.edu.cn (Z.Y.); xiaodongli@yic.ac.cn (X.L.); xlyin@yic.ac.cn (X.Y.); 2 College of Resources and Environment, University of Chinese Academy of Sciences, Beijing 100049, China; 3 School of Ocean, Yantai University, Yantai 264005, China; 4 Center for Ocean Mega-Science, Chinese Academy of Sciences, Qingdao 266071, China

**Keywords:** quantitative analysis, validation, steroid hormone, stressor, behavioral ecology

## Abstract

The quantification of steroid hormones of individual zebrafish (*Danio rerio*) provides perspective to understand endogenous hormone function. A UPLC–TOF–MS method was developed to provide a reproducible, sensitive, and efficient assay to determine the concentration of steroid hormones, including cortisol, testosterone, androstenedione, 11-deoxycortisol, 11-deoxycorticosterone, and 17-hydroxyprogesterone in whole-body homogenates of each zebrafish. Solid-phase extraction was used to sample matrix clean-up and acquired a recovery from 89.7% to 107.9%. The analytes were separated on an Aquity BEH C18 column using gradient elution. Mass spectrometric analysis was performed by single reaction monitoring (SRM) using positive electrospray ionization mode. The total running time was 6 min, which was greatly shortened compared with a previously reported method. The developed method exhibited excellent linearity for all the analytes, with regression coefficients higher than 0.99. The limit of detection varied between 0.1 and 0.5 ng/L and the limit of quantification was 0.5–1.7 ng/L for all analytes. The precision of the method was assessed on replicate measurements and was found to be in the ranges of 1.9 % to 6.6% and 4.3% to 8.6%, for intra- and inter-day analysis, respectively. This method was validated according to FDA guidance and applied to determine steroid hormone levels in the tissue homogenate of zebrafish acutely treated with caffeine and ethanol.

## 1. Introduction

**Figure 1:**
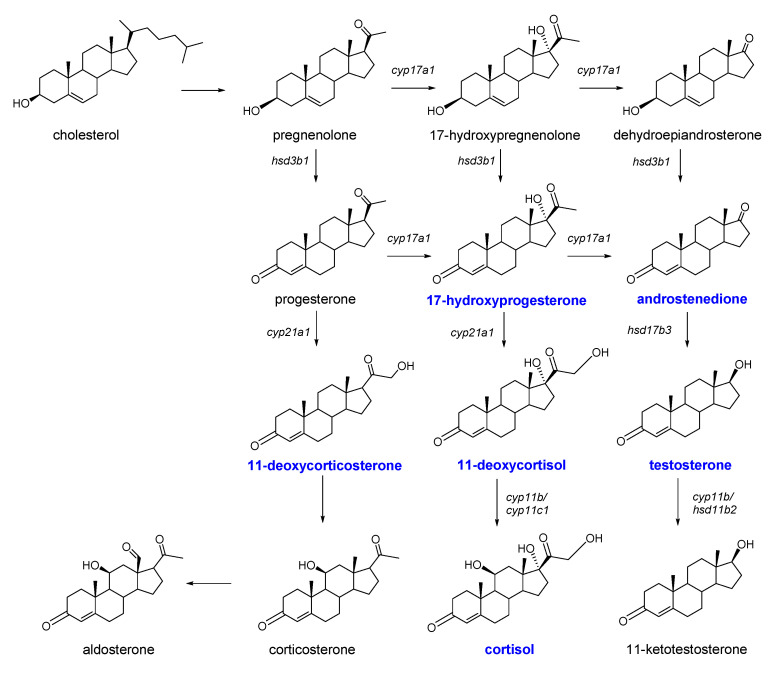
The plausible biosynthesis pathways of steroid hormones in a teleost [3]. The metabolites marked in blue are the analytes in this analysis method.

Zebrafish share many developmental aspects with their mammalian counterparts and many features in the endocrine system, including hormones, receptors, and signaling cascades [1,2]. Therefore, zebrafish has been broadly used as a model for developmental biology and endocrinology studies [3]. Steroid hormones are a class of endogenous metabolites involved in the steroidogenesis pathway, exerting profound impacts on ovarian development, oocyte maturation, and reproduction in fish and other vertebrate species [4]. In zebrafish, the pathway of steroidogenesis has been characterized and a plethora of steroid hormones have been shown to play similar roles compared to those in mammals (Figure 1) [3]. In this species, androgens regulate male sexual differentiation and behavior [5] and estrogens affect anxiety and shoaling behavior [6] in different gender and life stages [7]. The cortisol level in zebrafish is a crucial index to understand behavioral and physiological phenotypes of stress and anxiety [8]. In addition, other steroid hormones are also of great interest for psychobiological inquiries, including testosterone, dehydroepiandrosterone, and its sulfate ester, as well as progesterone. The selected six hormones are involved in different putative biosynthesis pathways (Figure 1), which may be helpful in discovering steroid function under acute stress.

The zebrafish also serves as a model laboratory species for testing endocrine-disrupting compounds in the environment to assess the potential ecological impacts [9,10]. Ste-roid hormones further elicit a behavioral response in conspecifics by acting as pheromones [11]. Steroid glucuronides, i.e., 17*α*,20*β-*dihydroxy-4-pregnen-3-one, testosterone, androsterone, epiandrosterone, and 5*α*-androstane-3*α*,17*β*-diol glucuronide, may function as ovulation-inducing pheromones in female zebrafish [12]. In addition, exogenous hormones can affect endocrine and developmental patterns in fish regardless of the level of dependence on endogenous steroids [13]. To delineate the correlation between stimuli and anxiety behavior patterns, quantitative analyses of cortisol in zebrafish whole body have been employed [14].

As a general protocol, the cortisol level in individual fish is measured by the enzyme-linked immunosorbent assay (ELISA) [15], which was initially developed for human saliva cortisol determination [16]. Usually, steroids are analyzed by immunoassay [17,18,19], which is the most widely used assay in clinical laboratories. Such methods include colorimetric ELISA assay [17], chemiluminescent immunoassay [18], electro-chemiluminescence immunoassay, immunochromatographic test, or sensors and immunosensors [19]. The immunoassays display disadvantages; for instance, the antibodies used in immunological tests are prone to interference by cross-reacting steroids, resulting in false-positive data. To overcome the drawback of cross reactivity with similar analytes, standardization issues between laboratories, and sensitivity issues, editors from the *Journal of Clinical Endocrinology and Metabolism* recommended to avoid using immunoassays, and instead use MS for the measurement of sex steroids [20]. Moreover, immunoassays are time consuming and costly, because individual analytes must be tested on a specific assay kit individually. The less commonly measured steroid assays, such as 11-deoxycorticosterone, aldosterone, 17-hydroxyprogesterone, dehydroepiandrosterone, and dihydrotestosterone, are all readily measured using LC–MS/MS but are not available on main laboratory immunoassay platforms [21]. Determination of cortisol in serum by immunoassay suffers with interference from cortisol precursors, whereas prednisolone, prednisone, cortisol, and cortisone can all be selectively analyzed by LC–MS/MS without cross reactivity [22].

Mass spectrometry has been reported as the most sensitive and specific technique for the quantitative analysis of minute metabolites, and recently LC–MS/MS-based methods have become the most reliable assays to simultaneously quantify several steroid hormones [23]. The great advantage of LC–MS/MS is the ability to simultaneously measure several different steroids to produce multiplexed test channels. Mass spectrometry uses a variety of ionization techniques, including fast-atom bombardment (FAB), matrix-assisted laser desorption ionization (MALDI), and electrospray ionization (ESI), in combination with time-of-flight (TOF) and triple quadrupole (QQQ) tandem mass spectrometry [24]. The most used method is a triple quadrupole mass spectrometer [25,26,27], which allows quantitative analysis of target steroid hormones in samples. Jensen et al. developed a method based on liquid–liquid extraction (LLE) followed by liquid chromatography coupled with electrospray tandem mass spectrometry (LC–ESI–MS/MS) for simultaneous determination of salivary melatonin, cortisol, and testosterone [28]. Montskó et al. established methods for determining the concentrations of serum total, serum free, and salivary cortisol based on solid-phase extraction (SPE) and the application of high-performance liquid chromatography coupled with high-resolution ESI–TOF mass spectrometry [29]. Recently, Nouri et al. developed a robust method for the simultaneous quantification of 14 steroid hormones in fish homogenates by LC–MS/MS [30], in which the specificity is achieved by the fragmentation of hormones, requiring a multiple reaction monitoring (MRM) transition. Büttler et al. compared eight routine LC–MS/MS methods for the simultaneous measurement of testosterone and androstenedione in serum and concluded that the observed differences in standardization should be taken into account [31]. Ray et al. developed a sensitive and specific method for the measurement of corticosterone, 11-deoxycortisol, 11-deoxycorticosterone, 17-hydroxyprogesterone, and progesterone in human serum and plasma by LC–MS/MS combined with differential ion mobility spectrometry [32]. Collectively, the analytical methods for hormone steroids by LC–MS/MS have been extensively investigated according to the research or clinical objectives. Our objective was to develop a sensitive and specific UPLC–MS-based hormone assay for zebrafish body homogenates using a QTOF high-resolution mass spectrometer. Specificity was achieved using accurate mass identification instead of an MRM transition.

## 2. Results and Discussion

### 2.1. Method Development

The quantitative method was validated for zebrafish homogenates on UPLC tandem TOF–MS according to the FDA Guidance for Industry-Bioanalytical Method Validation [33]. The baseline separation of six analytes was achieved within six minutes under optimized experimental conditions, such as mobile phase, pH, and flow rate.

**Figure 2:**
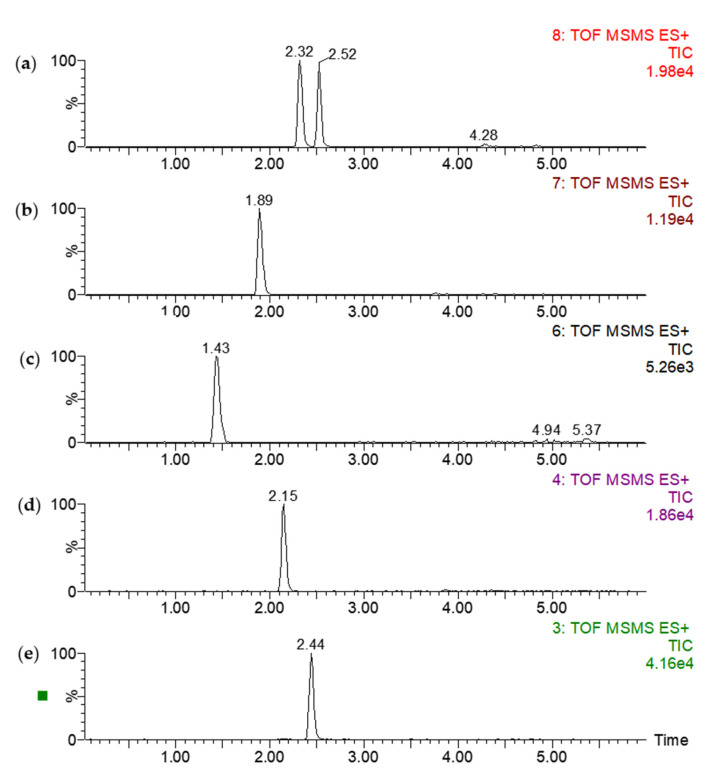
Chromatogram of six steroid hormone mixtures by LC–MS, (**a**) 11-deoxycorticosterone and 17-hydroxyprogesterone, (**b**) 11-deoxycortisol, (**c**) cortisol, (**d**) androstenedione, and (**e**) testosterone. Analyte standards: 200 ng/mL for each analyte. These steroids were detected by SRM mode and processed using MassLynx 4.1 software.

Chromatographic separation is crucial for the performance of an LC–MS analytical system, especially when applied to measure analytes at low concentration ranges, such as steroid hormones in biological matrices. LC separation of steroid hormones in biological fluid has usually been performed using C18 reverse-phase columns coupled with UPLC, which exhibits more advances in narrowing chromatographic peaks with higher resolution and shortened chromatographic run times. The first validated LC–MS/MS assay of salivary cortisol was reported using a Genesis C8 (2.1 mm × 20 mm) column with a particle size of 4 μm in 5 min by Jönsson et al. in 2003 [34]. The quantitative method developed on HPLC column (RP-18e, 4.6 mm × 50 mm) extended the running time to 20 min and resulted in the co-elution of cortisol and cortisone at 2.4 min [23]. Recently, the method was validated by using UPLC coupled with a C18 column (2.1 mm × 100 mm, 3.5 μm) for fish homogenates, with a total running time of 15 min [30]. In this developed method, a UPLC-specific reversed-phase C18 BEH column (2.1 mm × 50 mm) with a small particle diameter of 1.7 µm was chosen to achieve fast separation. Comparing the retention time of the analytes in this developed method with those in the literature, the retention time for cortisol is 1.43 min and testosterone is 2.44 min in our developed method, while the retention time for cortisol is 7.2 min and testosterone is 9.5 min in the literature [30]. Collectively, the shorter length of the column and smaller particle diameter of the stationary phase allowed a shorter running time for each sample. Overall, the developed analysis method achieved the separation of cortisol (1.43 min), testosterone (2.44 min), androstenedione (2.15 min), 11-deoxycortisol (1.89 min), 11-deoxycorticosterone (2.32 min), and 17-hydroxyprogesterone (2.52 min) in the total 6 min running time (Figure 2). Based on the currently available data, UPLC coupled with a small-particle-size column provides an efficiency development for steroid hormones analysis in biological matrices.

Retention time, order of elutes, and peak intensity were affected by mobile phases with different pH values. In previous reports, the steroid hormones were separated by mobile phase with ammonium fluoride [30], formic acid [32], or ammonium formate [35] as a buffer. In our developed method, the formic acid (0.1%) and ammonium acetate (10 mM) resulted in the most symmetric peak shape. Ammonium acetate can function as a chromatographic ion-pairing agent and is also compatible with the LC–MS detection system. Increasing the ammonium acetate to 20 mM resulted in distorted peaks for all analytes.

Positive ion single mass spectra of steroid hormones and tetra-deuterium-labeled cortisol have been reported previously using atmospheric pressure chemical ionization (APCI) [36] and atmospheric pressure photoionization (APPI) [37]. Specifically, the electrospray ionization (ESI) source has frequently been adopted as an ion source in conjugated hormone analyses using LC–MS. ESI is generally considered to yield better efficiency of ionization for steroid hormones than APCI [38]. In previous reports, most of the protocols for quantitative analyses of steroid hormones used the ESI in positive mode, although for some steroids negative mode has been used [39]. In our validated method, the ESI in positive mode was chosen according to the assessment of the chromatographic peak intensity of each analyte.

The quantitative analysis of steroid hormones on a QQQ mass spectrometer shows high sensitivity and specificity, as well as a large dynamic range. The acquisition was achieved using SRM [29], multiple reaction monitoring (MRM) [30], or multistage fragmentation (MS/MS/MS) modes [40]. In contrast with QQQ MS systems, the TOF–MS systems have been traditionally used for screening-type analyses rather than fully quantitative work. Recently, TOF–MS technology has gained the advantage of increased applicability for quantitative work, by the increase in the linear (dynamic) range and mass resolution of the instruments [41]. The most used Q-TOF or Orbitrap mass system possess a dynamic range of ≥4 orders of magnitude, a mass resolution of ≥30,000 (full width at half maximum), and a mass accuracy of <5 ppm. Use of Q-TOF–MS instruments for quantitative analyses is a feasible approach.

The analytes with identical mass to charge (*m*/*z*) hampered the application of Q-TOF in quantitative analysis. However, the advances of separation in UPLC can offset the drawback. In the developed method, the analytes 11-deoxycorticosterone and 17-hydroxyprogesterone had an identical *m*/*z* (Figure 2a). We optimized the gradient of mobile phase, flow rate, and column temperature, resulting in a baseline separation of 11-deoxycorticosterone (2.32 min) and 17-hydroxyprogesterone (2.52 min) (Figure 2a).

Solid-phase extraction (SPE) offers variability and high purification efficiency. In bio-analysis, the pre-treatment by SPE can eliminate the interference of contamination. Meanwhile, baseline separation on chromatography could avoid interference in the biological matrix. The cartridge with Oasis HLB sorbent has been exploited in the developed method due to its enormous potential for the extraction of compounds with high polarity [42]. The cartridge provided high recoveries (Table 1), consistent with the reported ability to capture acidic and neutral analytes across a wide polarity [43].

### 2.2. Calibration and Method Validation

The calibration curves were linear in calibration ranges of 0.3–200 ng/mL for all analytes. Although Koal et al. [23] reported the linear range of cortisol to reach 1000 ng/mL, this high concentration was not chosen due to its intensity reach of 8 × e^8^ in chromatography. The correlation coefficients for all the standard calibration curves were higher than 0.998 (Table 2).

According to the FDA concerning the validation of bioanalytical methods, the limit of detection (LOD) and limit of quantification (LOQ) determination method is the signal to noise ratio, which has been commonly used. The LOD for the individual analyte was determined at the lowest concentration, showing a signal to noise ratio (S/N) superior to 3, and ranged from 0.1 to 0.5 ng/mL (Table 2). The LOQ for the individual analyte was determined at the lowest concentration, showing a signal to noise ratio (S/N) superior to 10, and ranged from 0.3 to 1.7 ng/mL (Table 2), similar to the respective values reported recently [41].

The intra- and inter-day accuracy and precision were evaluated using 3 QC concentrations distributed throughout the calibration range for each analyte (Table 3). Intra-day (inter-day) accuracy (*n* = 5) ranged from 94.7 to 100.8% (92.2%–106.3%). For the precision of analytes, intra-day (inter-day) variation (*n* = 5) ranged from 1.9% to 6.6% (4.3–8.6%). The precision and accuracy for all analytes were more than 85%, which was within the interval set by the FDA concerning the validation of bioanalytical methods. Therefore, the developed method yields excellent reliability and reproducibility. Furthermore, the stability of stock solutions under storage conditions and the stability of extracted biological samples in the autosampler were tested. Standards were stable for at least 1 month in the −20 °C freezer, and 48 h in the 4 °C autosampler (data not shown).

### 2.3. Recovery and Matrix Effects

The recovery of the sample preparation procedure was evaluated by comparing each analyte’s area of calibration to the matrix study solution at the same concentration for 3 QC levels. The recovery rates ranged from 89.7% to 107.9% (Table 1). Each of the steroid hormones exhibited high recovery rates after the clean-up step, which is in accordance with the acceptable range (80–120%) set by the guidelines. The matrix effect was evaluated by comparing the ratio of the measured peak area in the spiked homogenate supernatant after the extraction to the peak area in the standard solution for 3 QC levels of each analyte (Table 1). The value of >0% indicates matrix enhancement and a value <0% indicates matrix suppression. In the developed method, the matrix effects ranged from −8.19% to 9.43%, which is in the range −20% < % matrix effect < +20% [44]. Thus, the matrix is not considered significant, indicating that the sample preparation procedure is accurate and reproducible.

### 2.4. Quantitative Analysis of Steroid Hormones in Zebrafish Homogenates

**Figure 3:**
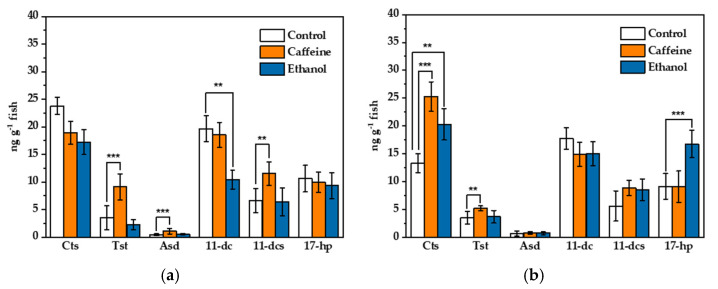
Steroid hormone levels in zebrafish male (**a**) and female (**b**) adults after caffeine (300 mg/L) and ethanol (1%) acute exposure (5 min), respectively. The level of each steroid hormone in individual fish was calculated by amount/body weight (ng/g). Data are presented as mean ± s.e.m. Two-way ANOVA (*n* = 10). Abbreviations: Cts, cortisol; Tst, testosterone; Asd, androstenedione; 11-dc, 11-deoxycortisol; 11-dcs, 11-deoxycorticosterone; 17-hp, 17-hydroxyprogesterone. *** indicates statistical significance at *p* < 0.01. ** indicates statistical significance at *p* < 0.05.

Physiological endpoints, such as steroid hormone levels, are valuable additions, parallel to behavioral observations in the study of stress. Although cortisol has been assumed as the product involved in a cascade of hormones, other steroid hormone levels (Figure 1) in the stress response have been ignored. The steroid hormone levels in zebrafish exposure to acute ethanol and caffeine are shown in Figure 3. Compared to the control, the testosterone and 11-deoxycorticosterone levels of the male group in caffeine exposure (Figure 3a) increased significantly. In the ethanol acute exposure, the 11-deoxycortisol levels of the male group (Figure 3a) showed a significant decrease of 47%. In the female group, the cortisol level in the caffeine treatment and the 17-hydroxyprogesterone level in the ethanol exposure increased significantly (Figure 3b), while the tissue levels of the remaining steroid hormones showed no significant changes.

The testosterone/cortisol ratio (T/C) may present an indicator of the response to acute stress [45]. The T/C ratios in the control male and female groups were 0.16 ± 0.09 and 0.32 ± 0.23, respectively, which were not significantly different (*p* = 0.207). However, the ratio of the male group under caffeine exposure was 0.49 ± 0.15, which was much higher than its counterpart in the female group (0.21 ± 0.05). In ethanol exposure (Figure 3a), the T/C ratios of the male and female groups exhibited no significant difference with the control group. The results suggest that testosterone levels under acute caffeine exposure at 300 mg/L show sexual differences. The T/C ratio change is indicative that hormonal axes regulate both testosterone and cortisol levels [46].

## 3. Materials and Methods

### 3.1. Chemicals and Reagents

Acetonitrile, methanol, ethanol, tricane (Sigma-Aldrich, St. Louis, MO, USA), formic acid, and ammonium acetate (Sigma-Aldrich, Darmstadt, Germany) were HPLC grade. De-ionized water was prepared using a Milli-Q system (Millipore, Billerica, MA, USA). The blank bovine plasma, and the steroid standards, 11-deoxycorticosterone, corticosterone, aldosterone, 11-deoxycortisol, cortisol, androstenedione, testosterone, and tetra-deuterated cortisol, were bought from Sigma-Aldrich (purity > 99%, USA). The caffeine was refined in-house from food supplementary capsules (Nutricost, Vineyard, UT, USA). The chemical structure and purity were confirmed by NMR (Bruker, Rheinstetten, Germany) and HR-MS (Waters, MA, USA) in our laboratory (See Supplementary Materials). The solid-phase extraction cartridges (HLB, 100 mg sorbent per cartridge) were purchased from Waters (Milford, MA, USA).

### 3.2. Animals and Housing

All fish used in this study were experimentally naive. A total of 100 adult male and female zebrafish (3–5 month old) were obtained from a commercial distributer; the average weight was 0.2 g and average body length was 2.1 cm (Pinduoduo App, Fuzhou, Fujian, China). All fish were acclimated to the laboratory environment for at least 10 days and housed in groups of 10–20 fish per 10 L tank in a zebrafish laboratory aquarium system (Haisheng, Shanghai, China). All tanks were filled with deionized water treated with a water filtration system (Haier, Qingdao, China) and spiked with sea salt to approximately 700 ppm. The room and water temperatures were maintained at 25–27 °C. Illumination was provided by ceiling-mounted fluorescent light tubes on a 12 h cycle (on: 08.00, off: 20.00). Fish were fed with brine shrimp (Jingdong APP, Beijing, China).

### 3.3. Preparation of Calibration Standards, Quality Control, and Internal Standard Solutions

The steroid hormone standards were separately dissolved in methanol individually (10 µg/mL). Stock solutions were combined and then serially diluted with commercially available blank plasma to produce standard solutions for a calibration curve (0.1, 0.2, 0.5, 1.0, 2.0, 5.0, 10.0, 20.0, 50.0, 100.0, 200.0 ng/mL for each compound). Quality control (QC) samples were prepared at three concentration levels, higher, middle, and lower limit of quantification, and abbreviated as HQC, MQC, and LQC, respectively, based on the dynamic ranges of the analytes (Table 2). Cortisol-d_4_ (Sigma-Aldrich) was used as the internal standard and dissolved in methanol to produce a 50 ng/mL IS solution. All stock solutions were sealed and stored at −20 °C until use. Before use, each solution was thawed at room temperature for 30 min.

### 3.4. Tissue Homogenization, Extraction, and Sample Preparation for Quantification

Briefly, the whole-body cortisol of each fish was extracted using the method for Chinook salmon, *Oncorhynchus tshawytscha*, eggs, and embryos, with modification [47]. The harvested fish were euthanized in 500 mg/L tricane on an ice bath, blotted on paper towels to remove excess water, immediately frozen in liquid nitrogen, and stored at −80 °C until analysis. All experimental procedures were approved by the Institutional Animal Care and Use Committee (Protocol # 001/2021). The whole zebrafish were thawed, weighed, homogenized, and centrifuged. Briefly, each defrosted fish was chopped into small pieces and placed in a centrifuge tube. Fish samples were homogenized individually at 60 Hz for 1 min, and successively centrifuged for 10 min at 12,000 rpm, 4 °C. The supernatants were pre-concentrated by SPE methods with the Oasis HLB Vac Cartridge. The sample was spiked with cortisol-d_4_ as an internal standard and loaded onto SPE cartridges pre-conditioned with 10 mL of methanol and 10 mL of H_2_O. Samples were passed through the cartridges at a rate of 1 mL/min using a Supelco vacuum manifold (Sigma-Aldrich Corp, Saint Louis, MI, USA), which allowed for the parallel extraction of up to 12 samples. Loaded cartridges were washed first with 30% methanol/water containing 2% acetic acid (5 mL) to remove interferences. Successively, they were eluted by 75% methanol/water containing 2% acetic acid (5 mL) and the eluates were collected [48]. The eluent was evaporated using a Cold Trap with Vacuum Centrifuge Concentrator (JM technology Co., Beijing, China), and reconstituted in 100 μL of methanol/H_2_O (55:45, *v:v*) for UPLC–MS analysis.

### 3.5. Quantitative Conditions by UPLC–TOF–MS

The LC–MS/MS system consisted of a Waters ACQUITY H-Class UPLC™ system connected to a Waters Xevo TQ-S triple quadrupole time-of-flight mass spectrometer (Waters Corp., Milford, MA, USA). The mobile phase consisted of water with FA, 0.1%, and 10 mM ammonium acetate as (A), and methanol with FA, 0.1%, and 10 mM ammonium acetate as (B). A Waters BEH C18 column (2.1 mm × 50 mm, 1.7 μm particle size) coupled with an Acquity UPLC™ column in-line filter kit (0.2 μm filter) was used. Separation was achieved using the following gradient program at a flow rate of 300 μL/min for 6 min at 18 °C: 45% A for 0.5 min, decreased to 5% A from 0.5 to 4 min, maintained at 5% A from 4.00 to 5.00 min, increased to 45% A from 5.0 to 5.01 min, and maintained at 45% A to 6 min for column equilibrium. The injection volume was 10 μL. The steroid hormones were detected by SRM mode and processed by Masslynx 4.1 software (Waters Corp.). The selected ion for each analyte was listed in Table 2. The UPLC effluent was introduced into the mass spectrometer with electrospray ionization in the positive mode. The ESI–MS parameters were set as follows: capillary voltage, 3.00 kV; extractor voltage, 5 V; source temperature, 150 °C; desolvation temperature, 400 °C; and desolvation gasflow, 800 L/h (N_2_, 99.9% purity). Data were collected in centroid mode with a scan range of *m/z* 50–500. The dwell time was set as auto, and the interscan delay was set at 20 ms. The internal standard was added to each sample to form 50 ng/mL concentration. Data acquisition was carried out by Masslynx 4.1 software and processed by QuanLynx (Waters Corp.).

### 3.6. Matrix Effects

The post-extraction addition method was employed for matrix effect evaluation. Briefly, ten samples were extracted according to the SPE procedures. After extraction, five samples were spiked with defined amounts of standard stock solutions, while the remaining five samples were kept as blank samples. Simultaneously, identical amounts of standard stock solutions and internal standards were spiked into five clean vials. All samples were then evaporated and dissolved in the mobile phase according to sample preparation and extraction, respectively. The matrix effect was calculated by the ratio of the measured peak area in the matrix spiked after the extraction (minus the peak area in the blank matrix sample) to the peak area in the standard solution.

### 3.7. Method Validation

All validation experiments were performed on body homogenate matrices. Method linearity was determined in standard solutions. The correlation coefficients were estimated through 1/χ least squares regression of the ratio of standard area vs. internal standard area. The limit of quantification threshold was determined by a signal-to-noise ratio of at least 10 (S/N ≥ 10), whereas LOD was estimated by a signal-to-noise ratio of at least 3 (S/N ≥ 3). Recovery parameters were evaluated with five replicates at each QC concentration.

According to the FDA biological sample guidance, accuracy and precision were determined with five replicates of each QC concentration within the same day (intra-day) and over five days (inter-day), respectively. The relative peak area for every target was expressed by the ratio of the target peak area over the peak area of the internal standard cortisol-d_4_. Accuracy was expressed by the ratio of the determined concentration over the spiked concentration. Precision was determined by the coefficient of variation within replicates.

### 3.8. Acute Exposure Manipulation

Briefly, the acute exposure assays followed the protocol as described [15]. The ethanol was administered acutely by placing individual zebrafish into 500 mL of 1% for 5 min (male *n* = 10, female *n* = 10). Caffeine (300 mg/L) was administered in a 1 L pre-treatment beaker for 5 min (male *n* = 10, female *n* = 10). The corresponding controls (male *n* = 10, female *n* = 10) which did not receive stimuli treatment during this time were housed in otherwise identical conditions. Following exposure testing, the animals were euthanized in 500 mg/L tricane (Sigma–Aldrich, USA) on an ice bath, and immediately dissected for further analysis.

### 3.9. Data Analysis and Statistics

A “marker table” comprising *m/z*, RT, and signal area values for each analyte was generated by QuanLynx module in MassLynx 4.1. Linear relationship calculations between signal areas and concentrations were adopted by weighted least squares regression. The data were exported into SPSS Statistics 24 for further multivariate analysis, with ANOVA for assessing significant differences between groups. The results are expressed in the form of mean value ± standard deviation, with significance evaluated using Tukey’s post hoc test and set at *p* < 0.05.

## 4. Conclusions

We developed a method based on the SPE approach, coupled with UPLC–TOF–MS, for the simultaneous quantification of steroid hormones in individual zebrafish homogenates. In this study, six steroid hormones were chromatographically separated in six minutes, and data were acquired in positive ion mode using the SRM method. The HLB cartridge provided excellent recoveries (89.7%–107.9%) for all analytes. The developed method was validated with respect to linearity, retention time, reproducibility, precision, and accuracy. Our findings reveal that cortisol and testosterone levels present a significant difference in acute caffeine exposure, and the T/C ratio may serve as an index to access the stress response. These results provide an integral understanding of the steroid hormone level changes in response to caffeine exposure. The method opens a new avenue to profile the steroid hormone surge in the biosynthesis pathway.

## Figures and Tables

**Table 1:** Mean extraction recoveries and matrix effect of the steroid hormones in tissue homogenates (*n* = 5).

Analyte	QC Level	Recovery (%)	Matrix Effect (%)
Cortisol	LQC	97.60	4.31
	MQC	97.68	7.61
	HQC	95.57	9.43
Testosterone	LQC	107.47	−8.19
	MQC	104.91	−3.12
	HQC	105.20	−3.00
Androstenedione	LQC	106.93	0.99
	MQC	107.92	−3.37
	HQC	107.28	−2.37
11-Deoxycortisol	LQC	92.53	8.68
	MQC	93.09	6.03
	HQC	92.07	7.50
11-Deoxycorticosterone	LQC	89.73	7.17
	MQC	91.12	6.89
	HQC	90.61	8.35
17-Hydroxyprogesterone	LQC	96.93	−1.68
	MQC	95.60	0.83
	HQC	95.17	0.79

**Table 2:** Optimized UPLC–MS parameters for each steroid hormone.

Analyte	*m*/*z*	RT	LR	R^2^	LOD	LOQ
Cortisol	363.2	1.43	0.3–200	0.9999	0.1	0.3
Testosterone	289.2	2.44	0.3–200	0.9998	0.1	0.3
Androstenedione	287.2	2.15	0.3–200	0.9999	0.1	0.3
11-Deoxycortisol	347.2	1.89	0.7–200	0.9999	0.2	0.7
11-Deoxy-Corticosterone	331.2	2.32	0.7–200	0.9997	0.2	0.7
17-Hydroxy-Progesterone	331.2	2.52	1.7–200	0.9998	0.5	1.7

*m*/*z* corresponds to (M + H)^+^. RT (min), retention time. RTs are derived from Figure 1. LR, linear range; *R^2^*, correlation coefficient; LOD (ng/mL), limit of detection; LOQ (ng/mL), limit of quantitation.

**Table 3:** Intra-day and inter-day accuracy and precision of the LC–MS method (n = 5).

Analyte	QC Level	Calculated Concentration (Mean ± SD, ng/mL)		Accuracy (DEV, %)		Precision (RSD, %)	
		Intra-Day	Inter-Day	Intra-Day	Inter-Day	Intra-Day	Inter-Day
Cortisol	LQC	15.1 ± 1.0	14.5 ± 1.3	100.8	96.7	6.6	8.6
	MQC	75.4 ± 3.5	79.7 ± 3.9	100.5	106.3	4.6	4.9
	HQC	148.9 ± 3.7	154.4 ± 4.3	99.3	102.9	2.5	2.8
Testosterone	LQC	14.2 ± 0.4	14.7 ± 0.6	94.7	97.8	2.8	4.1
	MQC	74.9 ± 2.3	75.1 ± 3.4	99.9	100.1	3.1	4.5
	HQC	143.6 ± 2.8	145.3 ± 6.2	95.7	96.9	1.9	4.3
Androstenedione	LQC	14.3 ± 0.6	13.8 ± 0.7	95.1	92.2	4.2	5.1
	MQC	74.9 ± 2.3	75.9 ± 4.8	99.9	101.2	3.1	6.3
	HQC	146.6 ± 4.5	146.4 ± 5.7	97.7	97.6	3.1	3.8
11-Deoxycortisol	LQC	14.6 ± 0.8	13.9 ± 0.9	97.5	92.7	5.8	6.5
	MQC	74.8 ± 2.3	76.0 ± 4.4	99.7	101.3	3.1	5.8
	HQC	144.5 ± 5.1	137.2 ± 5.5	96.4	91.5	3.5	4.0
11-Deoxy- Corticosterone	LQC	15.0 ± 0.6	15.1 ± 1.2	100.0	100.9	4.0	7.9
	MQC	74.9 ± 2.3	78.8 ± 2.7	99.8	105.0	3.1	3.5
	HQC	145.4 ± 5.8	144.4 ± 6.1	97.0	96.2	4.0	4.2
17-Hydroxyprogesterone	LQC	14.5 ± 0.7	14.6 ± 0.8	96.6	97.3	4.8	5.5
	MQC	74.8 ± 2.3	79.0 ± 3.6	99.8	105.3	3.1	4.8
	HQC	146.7 ± 6.7	144.0 ± 7.0	97.8	96.0	4.6	4.9

Note: nominal concentrations for LQC, MQC, and HQC were designated as 15 ng/mL, 75 ng/mL, and 150 ng/mL, respectively.

## Data Availability

Data are contained within the article or Supplementary Materials.

## Supplementary Materials

The following are available online, Figure S1: Chromatogram of caffeine refined from dietary supplements; Figure S2: High-resolution mass spectrum of refined caffeine; Figure S3: ^1^H NMR spectrum of refined caffeine; Figure S4: ^13^C NMR spectrum of refined caffeine; Table S1: Summary of LC–MS method for quantitative analysis of steroids.

## Sample Availability

Samples are not available from the authors.
