# Supplementary material for: UPLC–TOF–MS Method for Simultaneous Quantification of Steroid Hormones in Tissue Homogenates of Zebrafish with Solid-Phase Extraction
Source: Molecules. 2021 Oct 14;26(20):6213. doi: 10.3390/molecules26206213 (PMC8539474; doi:10.3390/molecules26206213)
Supplement: Supplementary file 1 [file molecules-26-06213-s001.zip › molecules-1400132-supplementary.pdf]

Supplementary Materials

UPLC–TOF–MS Method for Simultaneous Quantification of Steroid Hormones in Tissue Homogenates of Zebrafish with Solid-Phase Extraction

Yaxi Li [1,2], Zhi Yan [1,3], Xiaodong Li [1], Xiuli Yin [1] and Ke Li [1,4,*]

[1] Yantai Institute of Coastal Zone Research, Chinese Academy of Sciences, Yantai 264003, China; yxli@yic.ac.cn (Y.L.); yanzhistudy@s.ytu.edu.cn (Z.Y.); xiaodongli@yic.ac.cn (X.L.); xlyin@yic.ac.cn (X.Y.)
[2] College of Resources and Environment, University of Chinese Academy of Sciences, Beijing 100049, China
[3] School of Ocean, Yantai University, Yantai, 264005, China
[4] Center for Ocean Mega-Science, Chinese Academy of Sciences, Qingdao 266071, China
* Correspondence: kli@yic.ac.cn; Tel.: +86-535-210-8286

Table of Content

Figure S1. Chromatogram of caffeine refined from dietary supplements 1

Figure S2. High Resolution mass spectrum of refined caffeine 2

Figure S3. ^{1}H NMR spectrum of refined caffeine 3

Figure S4. ^{13}C NMR spectrum of refined caffeine 4

Table S1. Summary of LC-MS method for quantitative analysis of steroids…………5

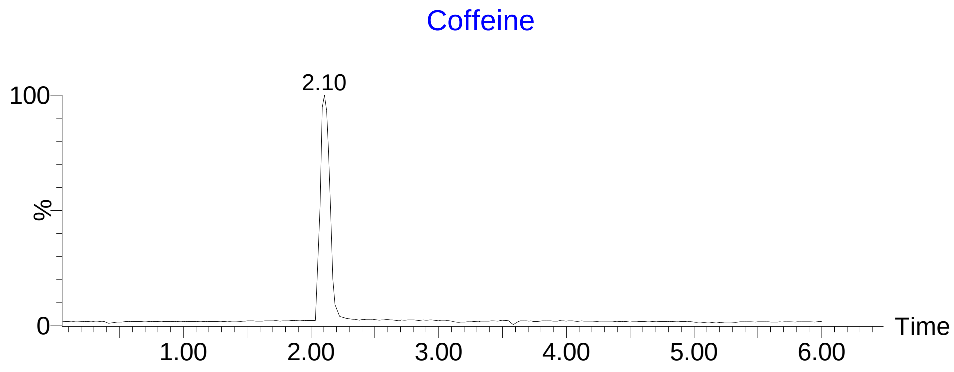

Figure S1. Chromatogram of caffeine refined from dietary supplements. The chromatographic separation was performed on Waters ACQUITY H-Class UPLC™ system connected to a Waters Xevo TQ-S triple quadrupole time of flight mass spectrometer (Waters Corp., Milford, MA, USA). The mobile phase consisted of water with FA, 0.1%, as (A) and methanol with FA, 0.1% as (B). A Waters BEH C18 column (2.1 mm×50 mm, 1.7 μm particle size) coupled with an Acquity UPLC™ column in-line filter kit (0.2μm filter) was used. A flow rate of 300 μL/min for 1 min at 18 °C: 95% A for 0.5 min, decreased to 5% A from 0.5 to 4 min, and then maintained at 5% A from 4.00 to 5.00 min, increased to 95% A from 5.0 to 5.01 min, and stayed at 95% A to 6 min for column equilibrium.

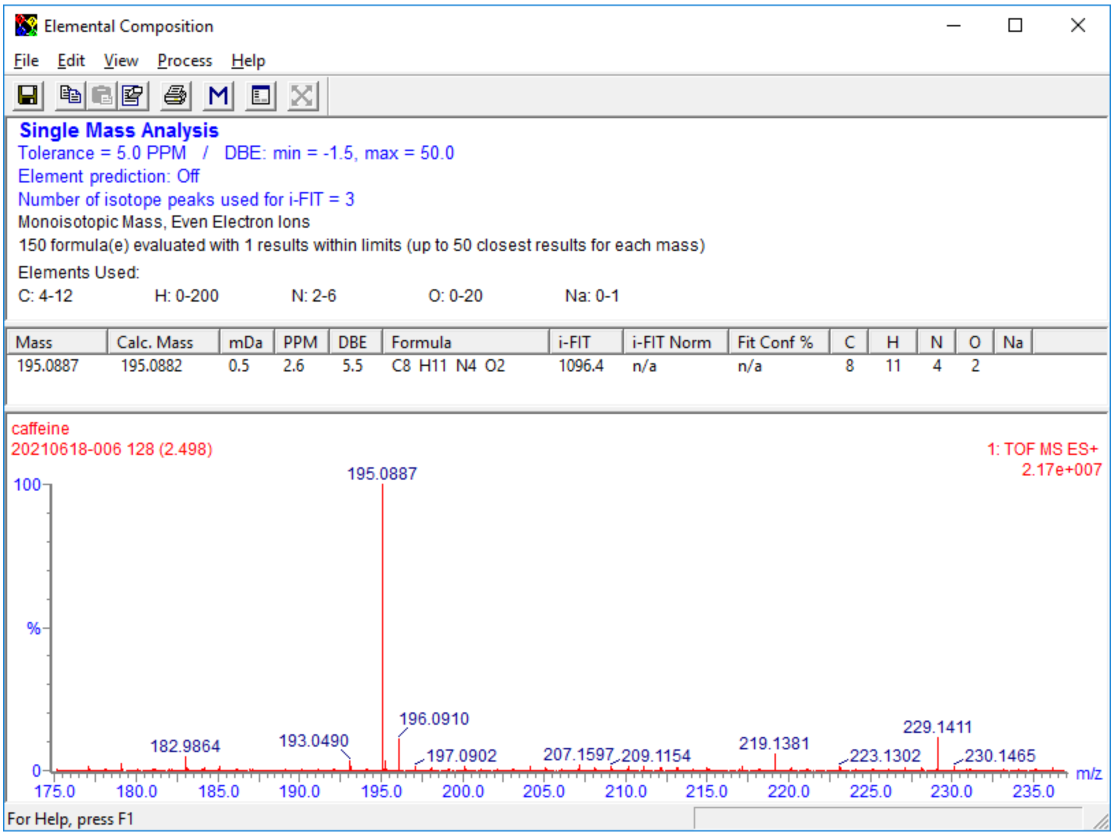

Figure S2. High resolution mass spectrum and elemental analysis of caffeine refined from dietary supplements. The refined caffeine was detected by positive mode and processed by Masslynx 4.1 software.

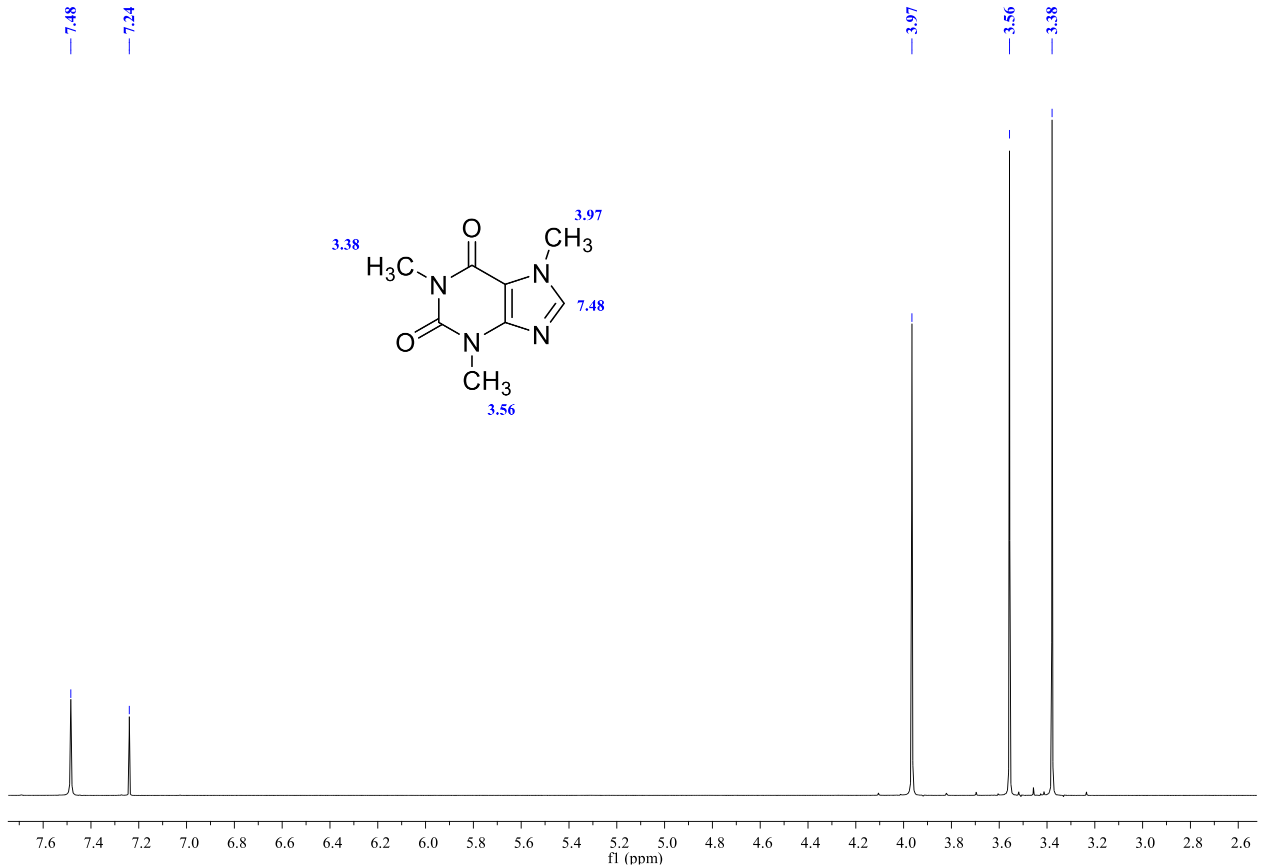

Figure S3. ^{1}H NMR spectrum of caffeine refined from dietary supplements.

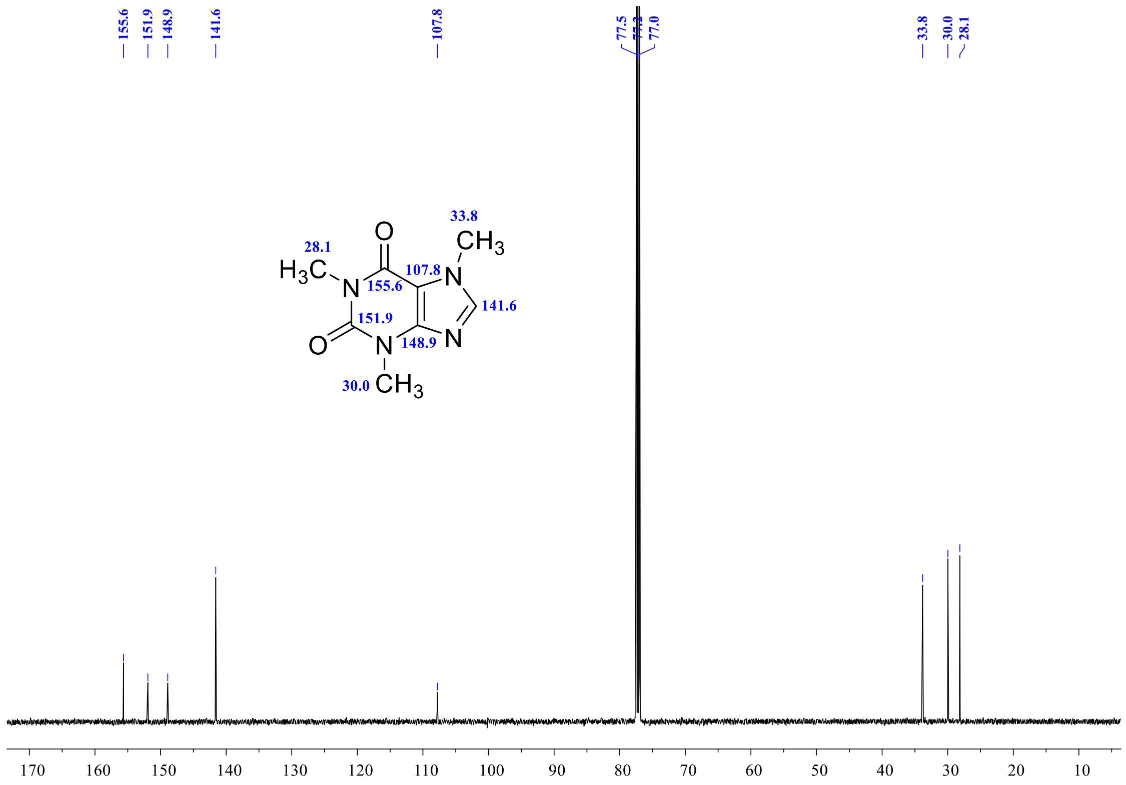

Figure S4. ^{13}C NMR spectrum of caffeine refined from dietary supplements.

Table S1. Summary of LC-MS method for quantitative analysis of steroids.

Analytes	Sample	instrument	Analytical column	Sample preparation	LOD	LOQ	Reference
Cortisol	Human salivary	Agilent HPLC series 1200-triple quadrupole mass spectrometer agilent 6430	Zorbax Eclipse XDB-C18 analytical column (4.6 × 50 mm, 1.8 μm particle size)	On-line SPE (Oasis HLB 1mL)	0.3 nmol/L;	0.55 nmol/L	[25]
Cortisol	Human salivary	Shimadzu UFLC tandem a QTRAP 5500 from AB SCIEX	Chromolith SpeedROD column (RP-18, endcapped, 50×4.6 mm) from Merck	On-line SPE (POROS 30×2.1 mm)	0.08 nmol/L;	0.08 nmol/L;	[26]
Cortisol	Human salivary	Agilent 1100 LC- API 4000 MS/MS (ACPI source)	Chromolith column (RP-18e, 100 mm × 4.6 mm)	On-line SPE (Oasis HLB 2.1 mm × 20 mm)	0.08 nmol/L	0.55 nmol/L	[27]
Cortisol	Human salivary	Agilent 1200 HPLC- Agilent 6460 QQQ equipped with a jet stream ESI ion source	a C18 2.1 mm × 50 mm 2.6 μm Kinetex column	liquid–liquid extraction (MTBE)	0.27 nmol/L;	None	[28]
testosterone	Human salivary	Agilent 1200 HPLC- Agilent 6460 QQQ equipped with a jet stream ESI ion source	a C18 2.1 mm × 50 mm 2.6 μm Kinetex column	liquid–liquid extraction	10.81 pmol/L	None	[28]
Cortisol	Blood (total)	Dionex Ultimate 3000 HPLC-Bruker micrOTOF high-resolution mass spectrometry	Kinetex C8 2.6 μm, 2.1× 100 mm analytical column	SPE (Strata-X (60 mg)	9 nmol/L	12.5 nmol/L	[29]
Cortisol	Whole fish body	Shimadzu UPLC- triple Quadrupole Linear Ion Trap	Eclipse plus C18 column, 2.1 × 100 mm, 3.5 μm	liquid–liquid extraction	0.012 ng/mL	0.025 ng/mL	[30]

		(QTrap 6500, AB sciex)		(MTBE)			
17OH-Progesterone	Whole fish body	Shimadzu UPLC- triple Quadrupole Linear Ion Trap (QTrap 6500, AB sciex)	Eclipse plus C18 column, 2.1 × 100 mm, 3.5 µm	liquid–liquid extraction (MTBE)	0.012 ng/mL	0.012 ng/mL	[30]
testosterone	Human serum	Acquity 2D-UPLC System - Xevo TQ-S tandem mass spectrometer	Kinetex Fluorophenyl-column (1.7 µm (2.1 × 100 mm)	2D-UPLC system with a BEH 300 C4-column (1.7µm, 2.1 × 50 mm)	None	0.05-0.30 ng/mL (nine methods)	[31]
androstenedione	Human serum	Acquity 2D-UPLC System - Xevo TQ-S tandem mass spectrometer	Kinetex Fluorophenyl-column (1.7 µm (2.1 × 100 mm)	2D-UPLC system with a BEH 300 C4-column (1.7µm, 2.1 × 50 mm)	None	0.05-0.35 (nine methods)	[31]
11-deoxycortisol	Human serum and plasma	HPLC-AB Sciex 5500 mass spectrometer	Kinetex C18, 50 × 3 mm, 2.6 µm	liquid–liquid extraction (MTBE)	0.16 nmol/L	0.30 nmol/L	[32]
11-deoxycorticosterone	Human serum and plasma	HPLC-AB Sciex 5500 mass spectrometer	Kinetex C18, 50 × 3 mm, 2.6 µm	liquid–liquid extraction (MTBE)	0.03 nmol/L	0.06 nmol/L	[32]
17-hydroxy progesterone	Human serum and plasma	HPLC-AB Sciex 5500 mass spectrometer	Kinetex C18, 50 × 3 mm, 2.6 µm	liquid–liquid extraction (MTBE)	0.08 nmol/L	0.19 nmol/L	[32]
